# Coral Mucus Microbial Community Change and Resistant Strategy Under UV Radiation: A Case from *Porites* sp. and *Favites* sp. Mucus Microbiome

**DOI:** 10.3390/microorganisms14061296

**Published:** 2026-06-09

**Authors:** Tianxiang Guo, Qun Jiang, Yaxing Liu, Chuanliang Wu, Zhiyong Li

**Affiliations:** 1 Hainan Research Institute, Shanghai Jiao Tong University, Sanya 572000, China; tx.guo@alumni.sjtu.edu.cn (T.G.); jiangq@sjtu.edu.cn (Q.J.); 2 State Key Laboratory of Microbial Metabolism, Life Sciences and Biotechnology, Shanghai Jiao Tong University, Shanghai 200240, China; 3 Sanya Coral Reef Ecology Institute, Sanya 572000, China; 18208983695@139.com (Y.L.); 52271300054@stu.ecnu.edu.cn (C.W.)

**Keywords:** coral mucus, microbial community, microbial isolation, UV resistance

## Abstract

Coral mucus serves as a crucial defensive barrier for corals, where the mucus microbes play a vital role in the coral’s resistance to external stresses. However, detailed knowledge of the effect of UV radiation on coral mucus microbiome is limited, particularly regarding the UV resistance mechanisms of coral mucus microbes. This study investigates changes in the mucus microbial community and possible UV-resistant mechanisms of the mucus microbiota of *Porites* sp. and Favites sp. under UV stress using high-throughput sequencing, UV-resistant microbial isolation, and RT-qPCR analysis. Compared to the control, UV stress alters microbial community structure by reducing microbial diversity, e.g., the relative abundance of *Aquibacter*, *Agaribacter*, and *Oleibacter* in coral *Porites* sp., and the *Roseobacter* clade CHAB_I_5 lineage, *Roseivirga*, and *Nautella* in coral *Favites* sp. increase under UV stress. Meanwhile, it is indicated that the *Favites* sp. mucus microbiome is much more sensitive than the *Porites* sp. mucus microbiome. A total of 428 microbial strains belonging to 5 phyla, 7 classes, 15 orders, 23 families, and 47 genera were isolated from these two coral mucus species, with *Ruegeria* and *Rossellomorea* as the most abundant cultured taxa. *Pseudoalteromonas galatheae* strain P12 and *Limimaricola pyoseonensis* strains P2 and P9 have been proven to exhibit higher UV resistance by enhanced expression of *tyr*, *sod*, *gogat*, and *uvrC* genes, indicating that the UV resistance of coral mucus bacteria involves complex molecular processes, including upregulation of antioxidant enzyme expression and enhancement of melanin and glutamic acid biosynthesis. These findings enhance our understanding of coral mucus microbial ecological functions, particularly highlighting the coral mucus microbial UV resistance strategy.

## 1. Introduction

Coral reefs are characterized by high biodiversity and hold significant ecological value for the marine ecosystem [1]. Apart from zooxanthellae, scleractinian corals are recognized as ‘holobiont’, complex consortia that comprise the coral host (polyp) and its diverse symbionts, including tissue-, mucus-, and skeleton-associated microorganisms [2,3]. A study based on microborings produced in vivo provided a glimpse into the ancient coral-skeleton microbiome [4]. Moreover, the skeleton microbiome harbors crucial members such as the endolithic algae *Ostreobium* spp., which may provide an alternative source of photoassimilates and be essential for coral survival during bleaching events [5]. These symbiotic microorganisms play an important role in maintaining the stability and productivity of coral reef ecosystems [6]. The surface mucus layer (SML) on corals constitutes a complex microenvironment, serving as a crucial interface between corals and their external environment [7]. The SML microbiota, including algae, bacteria, fungi, archaea, and viruses, are essential components of coral holobionts and closely linked to coral health and environmental adaptability, playing vital roles in nutrient cycling, pathogen defense, and immune regulation [8]. Kemp et al. [9] found a consistent distribution of microbial communities in the mucus of *A. palmata*, distinct from the surrounding environmental seawater microbiota. Under normal conditions, beneficial microbes dominate the coral mucus, providing the first line of defense against environmental stress. However, environmental disturbances can alter the SML microbial composition, increasing the risk of coral diseases [6]. Research on coral bleaching has indicated that the SML is crucial for the adhesion of pathogens such as *Vibrio shiloi* [10] and that bacterial communities in mucus and tissue exhibit different responses to bleaching stress [11]. Moreover, it is well known that the rheological properties of coral mucus change significantly under bleaching conditions, potentially undermining its protective function [12]. Lee et al. [13] demonstrated that heat stress significantly alters the bacterial composition in the mucus and tissues of coral *Acropora muricata*, shifting from γ-Proteobacteria to α-Proteobacteria, with increased abundances of Cyanobacteria, Flavobacteria, and Sphingobacteria at higher temperatures. Hussien et al. [14] identified similarities and differences in the microbial communities of coral mucus across different sampling sites in the Red Sea, indicating that, while a core microbiome exists, factors such as anthropogenic pollution can disrupt the SML microbial community. Seasonal variations also impact the microbial community in coral mucus, with α-Proteobacteria and γ-Proteobacteria showing seasonal differences [15]. These studies collectively indicate the responses of coral mucus microbial communities to environmental changes [16,17,18].

Coral mucus plays a crucial role in protecting corals from UV radiation. Mycosporine-like amino acids (MAAs) are key UV-protective components in coral mucus, capable of absorbing harmful UV radiation in the 310–360 nm range, thereby safeguarding corals and their symbiotic algae [19,20]. Coral mucus contains various MAAs that provide not only physical protection but also chemical defense against external stress and oxidative damage, mediated by enzymes such as phenoloxidase [21]. It was reported that some bacteria can produce absorption peaks in the UVA range, suggesting their role in protecting corals from UV damage [22], which is crucial for coral survival amid global climate change and increased UV exposure [16,23]. These findings have sparked interest in “microbiome engineering,” which involves using probiotics to enhance coral stress resilience [24]. However, as Thatcher et al. [25] pointed out, the effectiveness of such probiotics hinges on their ability to establish and persist within the coral mucus microhabitat [25]. Therefore, studies on changes in the coral mucus microbial community under UV radiation and their resistance to UV radiation are valuable for protecting corals, and the isolation and study of UV-resistant microbial strains will highlight the potential roles of coral mucus microbes in enhancing coral resilience to UV radiation. With this aim, this study investigates changes in the microbial community of coral mucus in *Porites* sp. and *Favites* sp. from the South China Sea under UV stress. Meanwhile, UV-resistant microbes were screened and evaluated for their UV-resistant mechanisms by functional gene expression analysis.

## 2. Materials and Methods

### 2.1. Coral Sampling and Mucus Collection

**Figure 1:**
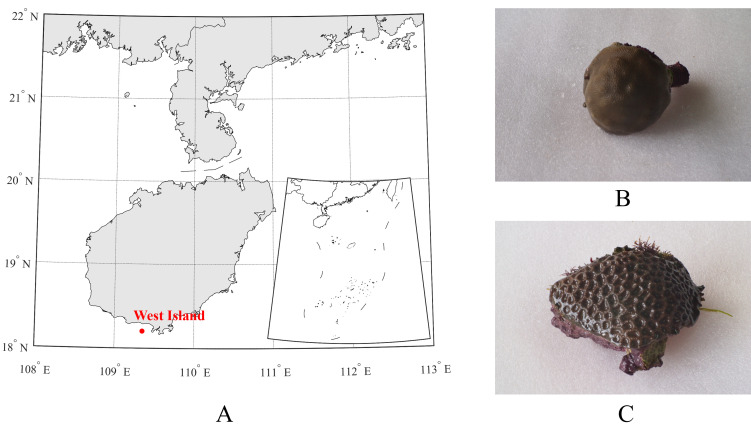
Geographical location of the coral sampling sites in West Island, Sanya (18°14′13.09″ N, 109°22′11.23″ E) (**A**); representative photographs of the sampled healthy *Porites* sp. (**B**) and *Favites* sp. (**C**) collected in July 2023. Species identification was performed based on skeletal morphological characteristics, including corallite structure and budding patterns. During the summer low tide period, samples were collected from the exposed intertidal zone.

Coral sampling was conducted in July 2023 (summer) in the intertidal zone of West Island, Sanya, Hainan (Figure 1A). The collected coral samples included two individuals of each *Porites* sp. (B) and *Favites* sp. (C) (a total of 4 individuals, with each parallel sample spaced by more than 100 m). The coral samples were immediately placed in insulated boxes filled with seawater and kept below 10 °C during transportation. The coral species were identified based on skeletal morphological characteristics, such as corallite structure and budding patterns, using standard taxonomic keys for scleractinian corals. To eliminate interference from seawater, each coral colony was rinsed with filtered, sterile seawater immediately after being removed from the tank. After thoroughly drying the coral surface with sterile gauze to remove excess moisture, the corals were exposed to the air for 10 min to stimulate mucus secretion under air-exposure stress. Using sterile pipettes in a sterile environment (pre-sanitized with 75% ethanol and conducted near an alcohol lamp), six 1 mL aliquots of mucus were collected from each individual and transferred into sterile centrifuge tubes, which were then divided into two groups: three aliquots served as the control group, and the remaining three were subjected to ultraviolet (UV) treatment.

### 2.2. UV Radiation Treatment and High-Throughput Sequencing Analysis

Mucus samples from two individuals of each species, *Porites* sp. and *Favites* sp., were used to compare microbial community composition. For each individual, three mucus tubes were assigned to the 3-day UV stress group (simultaneously exposed to visible light at 8000 lx, UVA365 at 200 μW/cm^2^ using a T8 UVA lamp with a peak emission at 365 nm and a spectral bandwidth of ~40 nm, and UVB297 at 30 μW/cm^2^ using a T5 UVB lamp with a peak emission at 297 nm and a narrow band spectral distribution; both lamps were from Shaanxi Antuo Biotechnology Co., Ltd., Xi’an, China). The other three mucus tubes served as the control group and were exposed only to visible light (8000 lx) without UV radiation. The visible light (8000 lx) was applied to both groups to simulate the natural light environment of corals. To ensure that any observed changes in the microbial community were specifically induced by UV radiation rather than experimental handling, the procedures of moisture removal and air exposure (which are necessary to stimulate mucus secretion) were standardized across both the control and experimental groups. All treatments were conducted in a constant temperature incubator maintained at 28 °C and 70% relative humidity to ensure consistent environmental conditions. A total of 24 samples (4 individuals × 6 tubes) were sequenced (Table S1). Mucus DNA was extracted using a modified CTAB method. Genomic DNA from the samples was extracted using a modified CTAB method combined with the TGuide S96 Magnetic Soil/Stool DNA Kit (TIANGEN Biotech (Beijing) Co., Ltd., Beijing, China). Specifically, the CTAB-based buffer and lysozyme were used for initial cell lysis at 65 °C, followed by DNA purification using the magnetic bead-based TGuide S96 platform according to the manufacturer’s instructions. The purity and concentration were confirmed via 1% agarose gel electrophoresis. The DNA samples were then diluted to 1 ng/μL. The DNA library was then constructed for the V4–V5 region of the 16S rRNA gene and sequenced on the Illumina Novaseq platform (Illumina, San Diego, CA, USA).

The V4–V5 hypervariable region of the 16S rRNA gene was amplified using the primers 341F (5′-CCTAYGGGRBGCASCAG-3′) and 806R (5′-GGACTACNNGGGTATCTAAT-3′) [26]. Paired-end sequencing (2 × 250 bp) was carried out on the Illumina NovaSeq platform. The DADA2 plugin in QIIME2 (version 2022.2; https://qiime2.org/, accessed on 2 June 2026) was used to perform strict quality control of the raw sequences [27]. Specifically, the forward and reverse reads were truncated at the positions where the median quality score dropped below 20, and low-quality sequences, including those with ambiguous bases or excessive expected errors, were removed. This denoising process produced accurate Amplicon Sequence Variants (ASVs), which served as the final feature units for all subsequent analyses. Taxonomic analysis was performed using the QIIME2 classify-sklearn naïve Bayes classifier against the Silva database (release 138.1; https://www.arb-silva.de/, accessed on 2 June 2026) [28]. Pan/core species analysis was performed to evaluate species richness in the samples and the stability of core species. To further assess whether the sequencing depth was sufficient to cover most microbial diversity, the ASV table was rarefied to the minimum sequence depth across all samples to normalize the sequencing effort, and Alpha diversity indices were calculated at different sampling depths using QIIME2. For microbial composition analysis, the feature table and taxonomic data were imported into the R language (version 4.3.1; https://www.r-project.org/, accessed on 2 June 2026) for detailed statistical analysis and graphical representation. Differential abundance was identified using the DESeq2 package (version 1.40.2; http://www.bioconductor.org/packages/release/bioc/html/DESeq2.html, accessed on 2 June 2026), with significance defined as |log2(fold change)| > 1 and an adjusted *p*-value (Benjamini–Hochberg) < 0.05, and volcano plots were created to illustrate species distribution and differences between samples.

### 2.3. Microbial Isolation, Identification, and UV-Resistant Strain Screening

Four types of culture media, such as seawater 2216E, R2A, GYP, and ST, were prepared using artificial seawater (ASW) to maintain appropriate salinity for marine microbial growth and used for coral mucus microbial isolation [29,30,31,32,33]. Mucus samples were collected from four coral colonies, with three biological replicates per colony. These samples were subsequently diluted and inoculated onto the four ASW-based media, followed by a 14-day cultivation period at 26 °C. Then, microbial colonies were selected for streaking on plates to obtain pure cultures. DNA was extracted using a commercial kit (TGuide Bacterial Genomic DNA Kit, Cat. No. OSR-M502, TIANGEN Biotech (Beijing) Co., Ltd., Beijing, China), and the 16S rRNA gene fragments were amplified with universal primers 27F and 1492R [34]. The 16S rRNA gene sequences were analyzed by the MegaBLAST algorithm using “Nucleotide BLAST (BLAST+ software package; v2.15.0+)” with the “16S ribosomal RNA sequences (Bacteria and Archaea)” database. Sequence similarity ≥99% and coverage ≥95% were used as criteria for species identification, with E-value <1 × 10^−50^ considered significant matches.

During screening for UV-resistant strains, two distinct strategies were employed depending on the intensity of UV stress. The screening was carried out in a pre-sterilized clean bench, with samples placed at a vertical distance of 40 cm from the UV light source. In the primary screening stage, both intense UVC and milder UVA/B stress were used to broadly identify potential candidates. Specifically, a relative growth rate (RGR) strategy under UVA/B was employed to identify strains producing inducible UV-protective compounds, while UVC exposure was used to pre-select strains with basic tolerance. Subsequently, in the secondary screening and quantitative resistance evaluation, the UV survival rate (USR) was used solely to assess the strains’ intrinsic resistance to intense UVC (254 nm, 107 µW/cm^2^) for 50 s, 100 s, 150 s, and 200 s. The UV dose (fluence, H) at which the survival rate decreases to 10% was calculated using the formula H = P × t, where P is the radiation power density (μW/cm^2^), and t is the irradiation time (s). The resulting values were converted to J/m^2^ to ensure comparability with existing literature [35]. In parallel, a relative growth rate (RGR) strategy under milder UVA/B stress was used to identify strains capable of producing inducible UV-protective compounds. Under this unified flux-based framework, the radiation power density (P) for UVC was 107 μW/cm^2^, while the specific irradiation times (t) were adjusted to achieve the desired UV doses (fluence) for D10 calculation.

### 2.4. UV Resistance-Related Function Gene Analysis

The isolated bacterial strain was inoculated into six culture tubes, divided into groups A and B, with three tubes in each group. For each biological replicate, three technical replicates were performed to ensure experimental reproducibility. Group A (control) was cultured in the dark at 26 °C under constant conditions, without UV stress, for 7 days. Group B was cultured under the same temperature conditions but with mild UV stress using simultaneous UVA (365 nm, 200 μW/cm^2^) and UVB (297 nm, 30 μW/cm^2^) for 7 days. This milder stress condition was selected to simulate the natural coral environment and investigate the expression of UV-responsive functional genes without causing excessive cell death. The reverse transcription quantitative PCR (RT-qPCR) method was used to analyze changes in the expression of the tyrosinase gene *tyr*, the glutamate synthase gene *gogat*, the excinuclease subunit C gene *uvrC*, and the superoxide dismutase gene *sod* in microbial strains following UV exposure (Table 1). To correct for variations across samples, *ftsZ* (a cell division protein gene) and *rpoD* (encoding the sigma factor subunit of bacterial RNA polymerase) were selected as reference genes. The expression stability of these internal controls under UV stress was pre-validated using the geNorm algorithm (integrated in qBase+ software version 3.4 or via reference URL: https://genorm.cmgg.be/, accessed on 2 June 2026), which showed consistent *Cq* values across all experimental groups (*M* < 0.5), confirming their suitability for normalization in this study [36,37].

For RT-qPCR, total RNA was extracted using the guanidine isothiocyanate method with a Bacterial Total RNA Extraction and Purification Kit (Sangon Biotech, Shanghai, China; Catalog No. B518655). RNA concentration and purity were measured using a NanoDrop spectrophotometer (A260/A280 > 1.8), and integrity was verified by agarose gel electrophoresis. Genomic DNA was removed during extraction using DNase I (Sangon Biotech). Subsequently, reverse transcription was performed using a one-step fluorescent quantitative reverse transcription kit (dye method) (One-Step Fluorescent Quantitative Reverse Transcription Kit, Sangon Biotech, Shanghai, China; Catalog No. B639277-0050) with 1 μg of total RNA to synthesize cDNA. After reverse transcription, quantitative PCR was carried out with the following program: 50 °C for 5 min, 95 °C for 3 min for pre-denaturation and enzyme activation; followed by 40 cycles of denaturation at 95 °C for 10 s and annealing, extension, and fluorescence signal acquisition at 60 °C for 30 s. The specificity of the products was confirmed by a single peak in the melting curve analysis. The amplification efficiency of all primers was verified to be within the range of 95–105% based on the standard curves generated from serial dilutions of cDNA. Moreover, the amplicon sizes for all target and reference genes were set between 100 and 200 bp to ensure optimal amplification efficiency. The expression levels of target genes were normalized to the reference gene (e.g., *ftsZ* and *rpoD*) and calculated using the 2^−ΔΔCT^ method as described by Livak and Schmittgen [38]. Each experiment was performed in three biological replicates, with three technical replicates for each biological sample. All data are presented as mean ± SD. Statistical differences were analyzed via Student’s *t*-test. Significance levels were set at * *p* < 0.05, ** *p* < 0.01, and *** *p* < 0.001. ns, not significant.

**Table 1:** Primers for the RT-qPCR analysis of UV resistance-related genes.

Gene Name	Primer Name	Primer Sequence	Reference
*ftsZ*	O1F	GCTAGCCCGGTTATCAAAGT	[39]
	O1R	AGTGTTGGCGCAGATGAA	
*rpoD*	O2F	GCCGAGATCAAGGACATCAA	[39]
	O2R	AGATCACCAGACGCAAGTTC	
*tyr*	t1F	TTCTTGCGACTGAGACCACC	[40]
	t1R	CGAATCCGACGCTACTACCT	
*gogat*	l1F	TCCACGCTAAGGGTAAACAT	[41]
	l1R	CACATTGCCACGCTCATCTA	
*uvrC*	u3F	GAGAATGGCGTGGCGTGTAT	[42]
	u3R	ATCTTGCGACCCGCTACTTC	
*sod*	k1F	CATACATTTCCCGCTCTGCC	[43]
	k1R	TCGCCATCTTTCACAACTAACC	

## 3. Results and Discussion

### 3.1. Coral Mucus Microbial Community Change Under UV Stress

**Figure 2:**
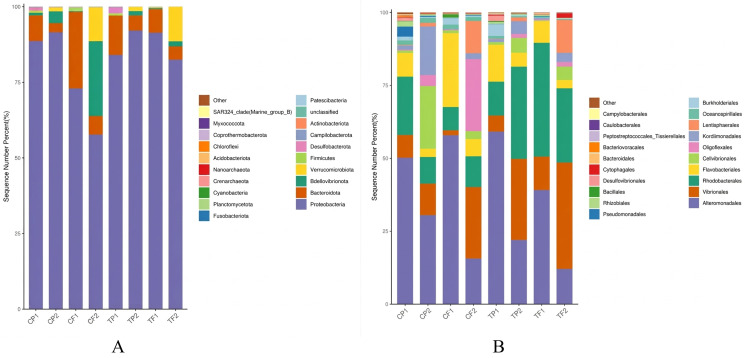
Coral mucus microbial composition comparison. (**A**): phylum level; (**B**): order level. C: control (normal light: 8000 lx); T: UV stress (simultaneous UVA365 200 μW/cm^2^ and UVB297 30 μW/cm^2^ for 3 days); P: *Porites* sp.; F: *Favites* sp.; the numbers 1 and 2 mean two individuals of the same coral species. Species 1 and 2 represent the full dataset from three parallel samples of the same individual of the same coral species; a total of 24 sequencing samples were included (Tables S1 and S2; Figure S1).

Based on the V4–V5 region sequencing of the 16S rRNA gene (Table S1; Figure S1), as Figure 2A shows that, at the phylum level for the control samples, the mucus microbial communities of corals *Porites* sp. and *Favites* sp. are mainly composed of Proteobacteria, this is consist with Kooperman et al. [44] that the Proteobacteria phylum is the most abundant taxa in coral mucus, indicating its widespread distribution and important ecological functions in coral mucus. In addition, Bacteroidota, Bdellovibrionota, and Verrucomicrobiota are also predominant taxa in the coral mucus. Archaea was also detected at lower relative abundance, including Crenarchaeota and Nanoarchaeota, as well as some OTUs unassigned to any phylum, indicating the presence of novel microbes in the coral mucus. Bdellovibrionota had a higher relative abundance in the control compared to a lower content in the UV stress group, suggesting that Bdellovibrionota may be sensitive to UV radiation [45]. Gammaproteobacteria and Alphaproteobacteria are the most dominant components (Figure S2), which is similar to the results of Lampert et al. [46]. Specifically, in UV-exposed samples, the relative abundance of Alphaproteobacteria is relatively higher, suggesting that Alphaproteobacteria may play a more important role in UV stress tolerance. Additionally, the study found that the relative abundance of Oligoflexia in the UV-exposed groups was lower than in the control, possibly because it is more susceptible to adverse environments [47].

At the order level (Figure 2B), Vibrionales accounts for a proportion of all samples, and may form a close symbiotic relationship with coral hosts [48]. In the UV-exposed group, the orders Campylobacterales and Rhizobiales are much more abundant than in the control, while the order Flavobacteriales is less abundant, indicating that UV exposure may have an impact on the composition of the coral mucus microbial community. The increase in the relative abundance of Campylobacterales and Rhizobiales indicates they are probably UV-tolerant, while the decrease in the relative abundance of Flavobacteriales may be due to their sensitivity to UV exposure [49,50].

**Figure 3:**
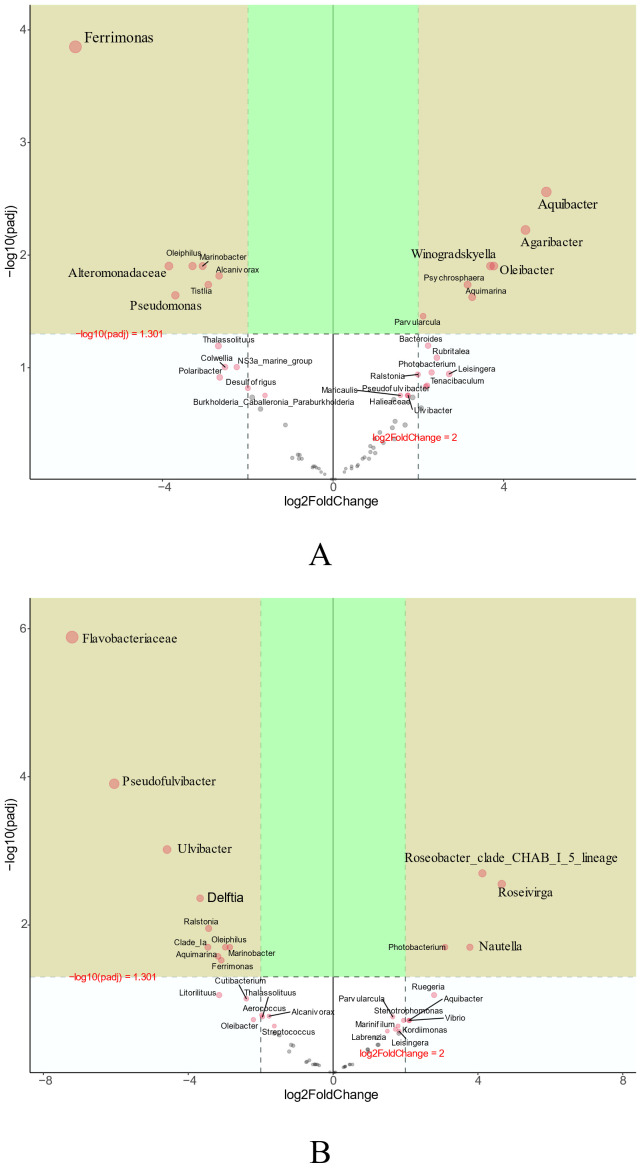
Comparative volcano plot of coral mucus microbes under UV stress (simultaneous UVA365 200 μW/cm^2^ and UVB297 30 μW/cm^2^ for 3 days) and the control (normal light: 8000 lx). The light yellow background indicates significantly enriched taxa (*p*adj < 0.05 and |log2FoldChange| > 2), the light green background indicates taxa with significant adjusted *p*-values but small fold changes, and the white background indicates non-significant taxa. Black dashed lines (with thresholds indicated by red text) represent the statistical boundaries (−log10(*p*adj) = 1.301 and log2FoldChange = 2). (**A**): *Porites* sp. mucus; (**B**): *Favites* sp. mucus.

Based on Figure 2A, for coral *Porites* sp., *Ferrimonas* and *Oleiphilus* have higher relative abundance in the control. In contrast, under UV exposure (synchronous UVA365 200 μW/cm^2^ and UVB297 30 μW/cm^2^ for 3 days), the relative abundance of *Aquibacter*, *Agaribacter,* and *Oleibacter* increased [51]. In the case of coral *Favites* sp. (Figure 2B), under UV exposure, the relative abundance of *Roseobacter* clade CHAB_I_5 lineage, *Roseivirga*, and *Nautella* increased. In contrast, dominant *Flavobacteriaceae*, *Pseudofulvibacter*, and *Ulvibacter* were detected in the control group. These microbiota with higher relative abundance in the UV stress group may be associated with stress resistance (Figure 3) [52,53].

As shown in Table S2, for *Porites* sp., no statistically significant differences were observed between the UV-treated (pB) and the control (nB) groups in Chao1 (152.42 vs. 142.69, *p* = 0.315), Ace (152.71 vs. 143.25, *p* = 0.310), Shannon (3.72 vs. 3.63, *p* = 0.729), Simpson (0.83 vs. 0.80, *p* = 0.469), or Faith’s phylogenetic diversity (20.96 vs. 18.14, *p* = 0.171). All *p*-values were greater than 0.05, indicating that *Porites* sp. exhibited a certain level of tolerance to the UV stress. For *Favites* sp., UV treatment resulted in a significant decline in alpha diversity compared to the control. Specifically, significant reductions were observed in Chao1 (156.35 vs. 115.15, *p* = 0.018), Ace (156.78 vs. 115.86, *p* = 0.017), Shannon (3.29 vs. 2.52, *p* = 0.043), and Faith’s phylogenetic diversity (16.78 vs. 12.58, *p* = 0.009). The Simpson index also decreased (0.74 vs. 0.60) but did not reach statistical significance (*p* = 0.075). This is consistent with the results of van de Water et al. [51], which showed that summer UVR reduced microbial community diversity. Environmental factors such as dissolved organic matter (DOM) and water turbidity can significantly regulate UV penetration. Moreover, UVA radiation can undergo photochemical transformation when it comes into contact with chromophoric DOM, initiating a photosensitization process. This results in the generation of reactive oxygen species (ROS), thus inducing an indirect oxidation process that intensifies oxidative damage to the microbiota. Based on the results above, UV stress reduced species richness, evenness, and phylogenetic diversity of the mucus microbiota in *Favites* sp., but had no significant effect on *Porites* sp. Based on these findings, the mucus microbiomes of corals *Porites* sp. and *Favites* sp. show different sensitivities to UV radiation, suggesting that coral species exhibit species-specific resistance to UV stress, with *Favites* sp. being more sensitive than *Porites* sp.

### 3.2. Coral Mucus Microbial Isolation and UV-Resistant Strain Screening

**Figure 4:**
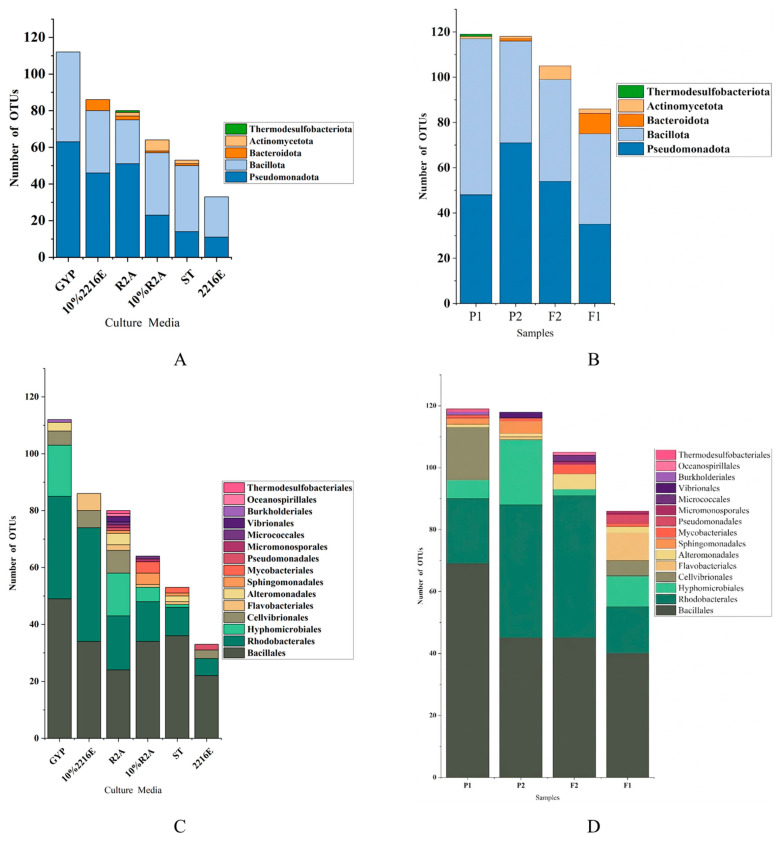
Diversity of cultivable bacteria from *Porites* sp. and *Favites* sp. mucus. (**A**,**B**): Taxonomic composition at the phylum level; (**C**,**D**): taxonomic composition at the order level. P and F represent *Porites* sp. mucus and *Favites* sp. mucus, respectively; the numbers 1 and 2 indicate two individuals of the same coral species.

Totally, 428 strains were isolated from the mucus of freshly collected corals *Porites* sp. and *Favites* sp. These isolated strains belong to Bacillales, Alphaproteobacteria, Gammaproteobacteria, Actinomycetales, Xanthomonadales, Betaproteobacteria, and Thermodesulfobacteria within phyla Proteobacteria and Firmicutes (Figure 4 and Figure S3). The marine agar GYP medium resulted in 54 strains of Alphaproteobacteria and 49 strains of Bacillales, followed by marine agar 10% 2216E medium, in which 40 strains of Alphaproteobacteria and 34 strains of Bacillales were isolated. For both coral species, *Bacillales* and *Rhodobacterales* are the predominant cultivable bacteria, i.e., 114 strains of *Bacillales* (accounting for 48.10%) and 64 strains of *Rhodobacterales* (accounting for 27.00%) in coral *Porites* sp., 85 strains of *Bacillales* (accounting for 44.50%) and 61 strains of *Rhodobacterales* (accounting for 31.94%) in *Favites* sp. This result is in agreement with that from the mucus of *Acropora digitifera* [54].

**Figure 5:**
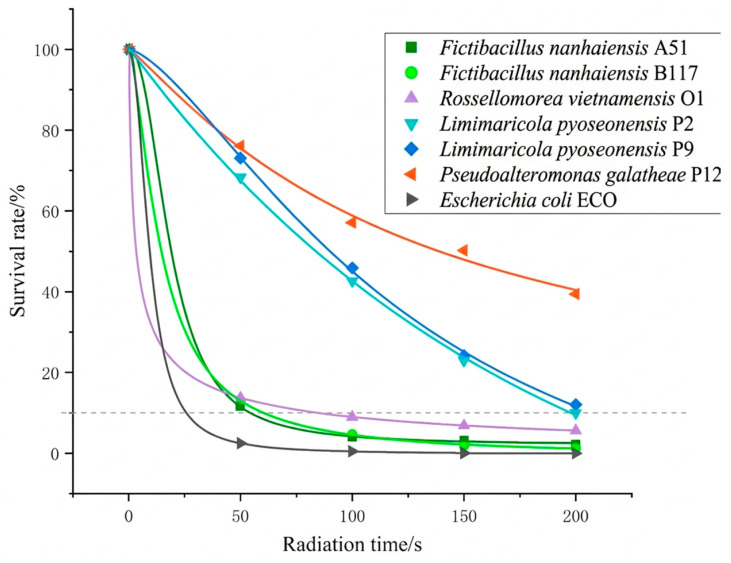
UV survival rate (USR) curve of isolated coral mucus bacteria under UV stress (UVC254, 107 μW/cm^2^). The dashed line indicates the threshold where the microbial survival rate is 10% (D10 value), rep-resenting the continuous exposure time required to achieve a 90% reduction of the initial bacterial population. Bacteria were exposed to UV irradiation for 50 s, 100 s, 150 s, and 200 s, respectively.

After 40 s of UV exposure (UVC254, 100 μW/cm^2^), most strains still survive. However, after 120 s of UV exposure (UVC254, 100 μW/cm^2^), the survival rate of most strains was close to zero; only 6 strains still showed a certain survival rate (Figure 5), which were identified as *Fictibacillus nanhaiensis*, *Rossellomorea vietnamensis*, *Limimaricola pyoseonensis*, and *Pseudoalteromonas galatheae* based on 16S rRNA gene (Table 2). The negative control, i.e., *Escherichia coli* (Eco), had the lowest D10 value, approximately 27.61 J/m^2^, due to its natural sensitivity to radiation [40]. In particular, *P. galatheae* strain P12 maintained a high survival rate of 92.01% after 120 s of UV exposure, with a D10 value of approximately 434.42 J/m^2^ (Figure 5; Table 2), suggesting that it has a strong UV resistance. Besides *P. galatheae* strain P12, *L. pyoseonensis* strain P9 had a D10 value of approximately 224.70 J/m^2^. The D10 value of *L. pyoseonensis* strain P2 was approximately 211.65 J/m^2^ (Table 2).

According to Table 2 and Figure 5, *Pseudoalteromonas galatheae* strain P12, *Limimaricola pyoseonensis* strains P2 and P9, *Fictibacillus nanhaiensis* strains A51 and B117, *Rossellomorea vietnamensis* strain O1 showed the strongest radiation resistance among the strains tested. *Rossellomorea vietnamensis*, *Limimaricola pyoseonensis*, and *Pseudoalteromonas galatheae* belong to the phylum Proteobacteria, while *Fictibacillus nanhaiensis* belongs to the phylum Firmicutes. Ravindran et al. [22] isolated UV-resistant bacteria from the mucus of *Porites lutea* and *Acropora hyacinthus*; most were Firmicutes, with the remainder Proteobacteria, consistent with the results of this study.

**Figure 6:**
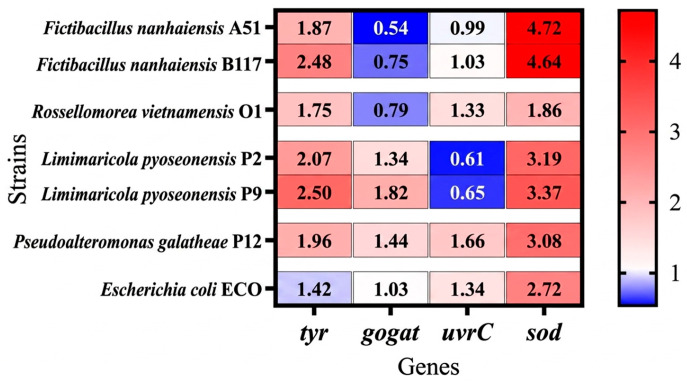
Gene expression change in isolated coral mucus bacteria under UV stress (simultaneous UVA365 200 μW/cm^2^ and UVB297 30 μW/cm^2^ for 7 days) compared to the control (normal light: 8000 lx).

**Figure 7:**
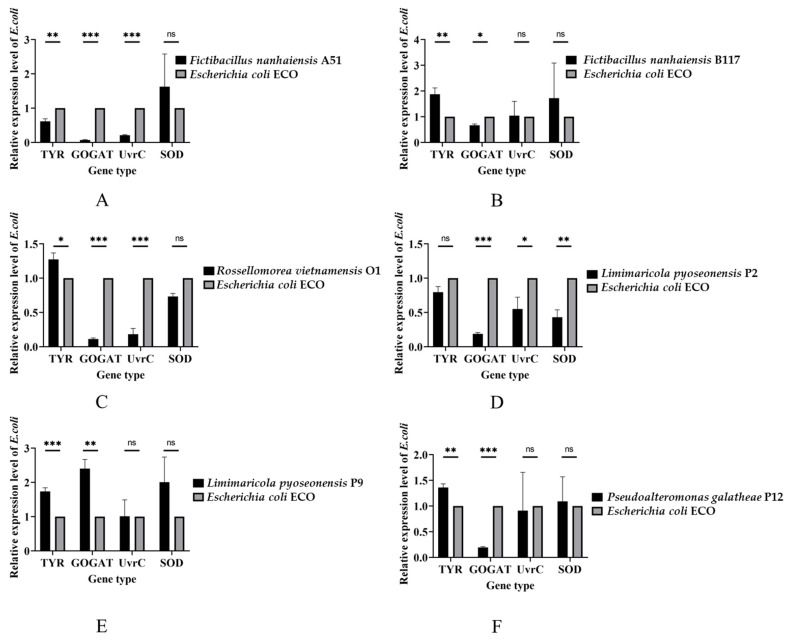
Gene expression comparison between coral mucus strains and *Escherichia coli* under simultaneous treatment of UVA365 200 μW/cm^2^ and UVB297 30 μW/cm^2^ for 3 days. (**A**) Expression in *Fictibacillus nanhaiensis* A51; (**B**) *Fictibacillus nanhaiensis* B117; (**C**) *Rossellomorea vietnamensis* O1; (**D**) *Limimaricola pyoseonensis* P2; (**E**) *Limimaricola pyoseonensis* P9; (**F**) *Pseudoalt-eromonas galatheae* P12. * *p* < 0.05, ** *p* < 0.01, *** *p* < 0.001; ns, not significant.

Then we measured the expression levels of four genes (*sod*, *tyr*, *gogat*, *uvrC*) in these six strains after UV exposure (UVA365 200 μW/cm^2^ and UVB297 30 μW/cm^2^ for 7 days), with *Escherichia coli* as the control (Figure 6). Overall, all bacteria tested (including *E. coli*) showed increased expression levels of the *sod* and *tyr* genes, while the *gogat* and *uvrC* genes showed no clear pattern. Enzymes such as tyrosinase (TYR) directly influence melanin synthesis. The upregulated expression of the *tyr* and *sod* genes in *Fictibacillus nanhaiensis* strains A51 and B117 was slightly higher than that in *E. coli*, suggesting that the *F. nanhaiensis* strains A51 and B117 enhanced melanin synthesis and reactive oxygen species degradation under UV stress (Figure 7), which may reflect a defense response of bacteria to oxidative stress and UV damage by upregulating the expression of *sod* and *tyr* genes. In addition, *L. pyoseonensis* strains P2 and P9, and *P. galatheae* strain P12, showed greater upregulation of the *gogat* gene expression than *E. coli*, suggesting that glutamic acid synthesis in these strains may be improved under UV stress.

Sansinenea et al. [55] found that melanin produced by *Bacillus subtilis* ELI52 enhances its resistance to UV radiation, maintaining the integrity and biological activity of proteins. Geng et al. [56] found that bacterial melanin effectively eliminates reactive oxygen species (ROS) following UVR exposure, helping prevent UVR-induced cell apoptosis. Du et al. [57] showed that when bacteria are subjected to UV stress, the transcriptional levels of the main reactive oxygen species (ROS) generation pathways (such as EMP) were downregulated, but the activity of the main degrading enzymes was upregulated. Therefore, bacteria may enhance their resistance to UV radiation by increasing melanin production and regulating the expression of antioxidant enzymes, thereby reducing ROS accumulation and oxidative stress. Glutamic acid is a precursor for glutathione, one of the most important antioxidants within cells, which can directly eliminate ROS. When UV radiation triggers ROS production, the cells’ demand for GSH increases sharply. Enhanced GOGAT activity can ensure an adequate supply of glutamate, thereby facilitating rapid GSH synthesis and helping cells resist oxidative stress. Thus, the activity level and functional integrity of GOGAT directly determine the logistical support capacity of the cell’s repair system after exposure to oxidative stress such as UV. These findings provide important clues to understanding the microbial response to UV stress and serve as a reference for the future development of UV-resistant microbial agents.

## 4. Conclusions

In summary, using the corals *Porites* sp. and *Favites* sp. as research models, this study comprehensively explores changes in the mucus microbial community under UV radiation and reveals the function and mechanism of coral mucus microbial UV radiation resistance. Though the mucus microbiota of corals *Porites* sp. and *Favites* sp. show different UV sensibility, UV radiation can change their mucus microbial community structure to some extent. Under UV stress, specific microorganisms in coral *Porites* sp., including *Aquibacter*, *Agaribacter*, and *Oleibacter*, and in coral *Favites* sp., including *Roseobacter* clade CHAB_I_5 lineage, *Roseivirga*, and *Nautella*, increase their relative abundance. In particular, *Pseudoalteromonas galatheae* strain P12 and *Limimaricola pyoseonensis* strains P2 and P9 exhibit greater UV resistance. The UV resistance strategy of coral mucus bacteria involves complex molecular processes, including the upregulation of antioxidant enzyme expression and enhancement of melanin and glutamic acid biosynthesis. These results expand our understanding of the microbial ecological function of coral mucus within the coral holobiont, particularly in protecting coral hosts from UV radiation. The findings have significant implications for reef management, especially in marine protected areas (MPAs). Monitoring these UV-sensitive or UV-resistant microbial indicators in MPAs can help evaluate habitat health under climate change. Moreover, the identification of these UV-resistant strains offers promising candidates for future microbiome engineering and probiotic applications aimed at enhancing coral resilience against environmental stress.

## Figures and Tables

**Table 2:** The UV (UVC254, 107 μW/cm^2^) resistance evaluation of isolated coral mucus bacteria.

Strain	Similarity	Similar Species	Time with a Survival Rate of 10% (s)	UV Dose for 10% Survival Rate (D10, J/m^2^)
A51	98.78%	*Fictibacillus nanhaiensis*	54.40	58.21
B117	98.96%	*Fictibacillus nanhaiensis*	60.20	64.41
O1	99.60%	*Rossellomorea vietnamensis*	85.00	90.95
P2	99.85%	*Limimaricola pyoseonensis*	197.80	211.65
P9	99.77%	*Limimaricola pyoseonensis*	210.00	224.70
P12	99.27%	*Pseudoalteromonas galatheae*	406.00	434.42
ECO	99.28%	*Escherichia coli*	25.80	27.61

## Data Availability

Sequence data supporting the findings of this study have been deposited in the NCBI BioProject database under accession PRJNA1430139. The 16S rRNA gene sequences of strains A51, B117, O1, P2, P9, and P12 with UV resistance ability have been deposited in NCBI under accession numbers PZ072079–PZ072084.

## Supplementary Materials

The following supporting information can be downloaded at https://www.mdpi.com/article/10.3390/microorganisms14061296/s1. Table S1: Quality control information of 24 coral mucus samples; Table S2: The α diversity index of 24 coral samples; Figure S1: Rank abundance curves of 24 samples (merged into 8 groups); Figure S2: Class-level analysis of two types of coral mucus under UV stress (simultaneous UVA365 200 μW/cm^2^ and UVB297 30 μW/cm^2^ for 3 days) compared to the control (normal light: 8000 lx); Figure S3: Diversity distribution of cultivable bacteria at the class level.
