# Supplementary material for: Coral Mucus Microbial Community Change and Resistant Strategy Under UV Radiation: A Case from Porites sp. and Favites sp. Mucus Microbiome
Source: Microorganisms. 2026 Jun 9;14(6):1296. doi: 10.3390/microorganisms14061296 (PMC13304099; doi:10.3390/microorganisms14061296)
Supplement: Supplementary file 1 [file microorganisms-14-01296-s001.zip › microorganisms-4271134-supplementary.pdf]

Supplemental materials:

Coral Mucus Microbial Community Change and Resistant Strategy Under UV Radiation: A Case from Porites sp. and *Favites* sp. Mucus Microbiome

Tianxiang Guo[1], Qun Jiang[1,2], Yaxing Liu[3], Chuanliang Wu[3], Zhiyong Li[1,2,3*]

[1] Hainan Research Institute, Shanghai Jiao Tong University, Sanya 572000, China; tx.guo@alumni.sjtu.edu.cn (T.G.); jiangq@sjtu.edu.cn (Q.J.)
[2] State Key Laboratory of Microbial Metabolism, Life Sciences and Biotechnology, Shanghai Jiao Tong University, Shanghai 200240, China
[3] Sanya Coral Reef Ecology Institute, Sanya 572000, China; 18208983695@139.com (Y.L.); 52271300054@stu.ecnu.edu.cn (C.W.)
* Correspondence: zyli@sjtu.edu.cn

Table S1. Quality control information of 24 coral mucus samples

Sample	input	filtered	percentage of input passed filter	merged	percentage of input merged	non-chimeric	percentage of input non-chimeric
CP1-1	93325	78663	84.29%	76131	81.58%	63266	67.79%
CP1-2	97625	82507	84.51%	76145	78.00%	63952	65.51%
CP1-3	90792	77237	85.07%	73529	80.99%	61081	67.28%
CP2-1	91912	76130	82.83%	72671	79.07%	59178	64.39%
CP2-2	93241	78195	83.86%	72593	77.86%	57213	61.36%
CP2-3	100901	82551	81.81%	77858	77.16%	63310	62.74%
CF1-1	102566	87451	85.26%	79905	77.91%	65765	64.12%
CF1-2	91496	78653	85.96%	76530	83.64%	65500	71.59%
CF1-3	91732	77548	84.54%	74070	80.75%	62029	67.62%
CF2-1	94197	79630	84.54%	74929	79.54%	64822	68.82%
CF2-2	94490	79451	84.08%	73854	78.16%	55908	59.17%
CF2-3	96125	79108	82.30%	73993	76.98%	62056	64.56%
TP1-1	90345	76351	84.51%	72666	80.43%	62702	69.40%
TP1-2	98561	86168	87.43%	83208	84.42%	72535	73.59%
TP1-3	80328	69704	86.77%	66314	82.55%	57378	71.43%
TP2-1	93058	79771	85.72%	74301	79.84%	55814	59.98%
TP2-2	93247	76633	82.18%	71906	77.11%	51477	55.20%
TP2-3	92635	79749	86.09%	73898	79.77%	52967	57.18%
TF1-1	90672	85975	94.82%	85120	93.88%	71678	79.05%
TF1-2	97343	82888	85.15%	77893	80.02%	61454	63.13%
TF1-3	94553	79965	84.57%	77420	81.88%	68399	72.34%
TF2-1	100448	95093	94.67%	84966	84.59%	57285	57.03%
TF2-2	92134	79473	86.26%	74842	81.23%	55116	59.82%
TF2-3	92880	78619	84.65%	73442	79.07%	58801	63.31%

Note: C: control (normal light: 8000 lx); T: UV stress (simultaneous UVA365 200 μW/cm² and UVB297 30 μW/cm² for 3 days); P: *Porites* sp. mucus; F: *Favites* sp. mucus; the numbers 1 and 2 mean two individuals of the same coral species; the numbers -1, -2, -3 after 1 and 2 mean three parallel samples of the same individual of the same coral species, i.e. totally 24 sequencing samples are included for two species of corals.

Table S2. The α diversity index of 24 coral samples

Sample	input	OTU	Ace	Faith_pd	Chao1	Shannon	Simpson	Coverage
CP1-1	63266	112	112.28	11.66	112.00	3.15	0.72	100.00%
CP1-2	63952	349	349.51	42.77	349.03	5.08	0.94	100.00%
CP1-3	61081	171	172.36	15.34	171.35	3.68	0.81	99.99%
CP2-1	59178	114	114.00	19.64	114.00	3.21	0.79	100.00%
CP2-2	57213	158	158.00	14.16	158.00	3.40	0.81	100.00%
CP2-3	63310	190	191.14	22.28	190.13	3.84	0.84	100.00%
CF1-1	65765	204	204.24	21.83	204.00	2.66	0.60	100.00%
CF1-2	65500	85	85.00	13.27	85.00	3.10	0.81	100.00%
CF1-3	62029	156	156.63	16.83	156.06	3.80	0.86	100.00%
CF2-1	64822	175	175.56	16.97	175.04	2.09	0.47	100.00%
CF2-2	55908	184	185.04	15.81	184.43	3.85	0.80	99.99%
CF2-3	62056	187	187.61	15.95	187.06	4.26	0.87	100.00%
TP1-1	62702	244	245.01	26.49	244.25	3.51	0.74	99.99%
TP1-2	72535	132	132.00	13.44	132.00	3.04	0.69	100.00%
TP1-3	57378	134	134.71	16.53	134.07	4.08	0.88	100.00%
TP2-1	55814	131	131.00	26.62	131.00	3.25	0.79	100.00%
TP2-2	51477	126	127.00	12.22	126.23	3.72	0.83	99.99%
TP2-3	52967	148	149.46	13.52	148.38	4.19	0.89	99.99%
TF1-1	71678	31	31.00	6.40	31.00	0.81	0.19	100.00%
TF1-2	61454	171	172.06	16.69	171.12	3.19	0.79	100.00%
TF1-3	68399	110	110.38	11.99	110.00	1.79	0.45	100.00%
TF2-1	57285	168	169.94	16.80	168.48	1.91	0.48	99.99%
TF2-2	55116	135	135.65	11.57	135.10	3.38	0.82	100.00%
TF2-3	58801	136	136.27	12.06	136.00	4.05	0.86	100.00%

Note: C: control (normal light: 8000 lx); T: UV stress (simultaneous UVA365 200 μW/cm² and UVB297 30 μW/cm² for 3 days); P: *Porites* sp. mucus; F: *Favites* sp. mucus; the numbers 1 and 2 ,mean two individuals of the same coral species; the numbers -1, -2, -3 after 1 and 2 mean three parallel samples of the same individual of the same coral species, i.e. totally 24 sequencing samples are included for two species of corals.

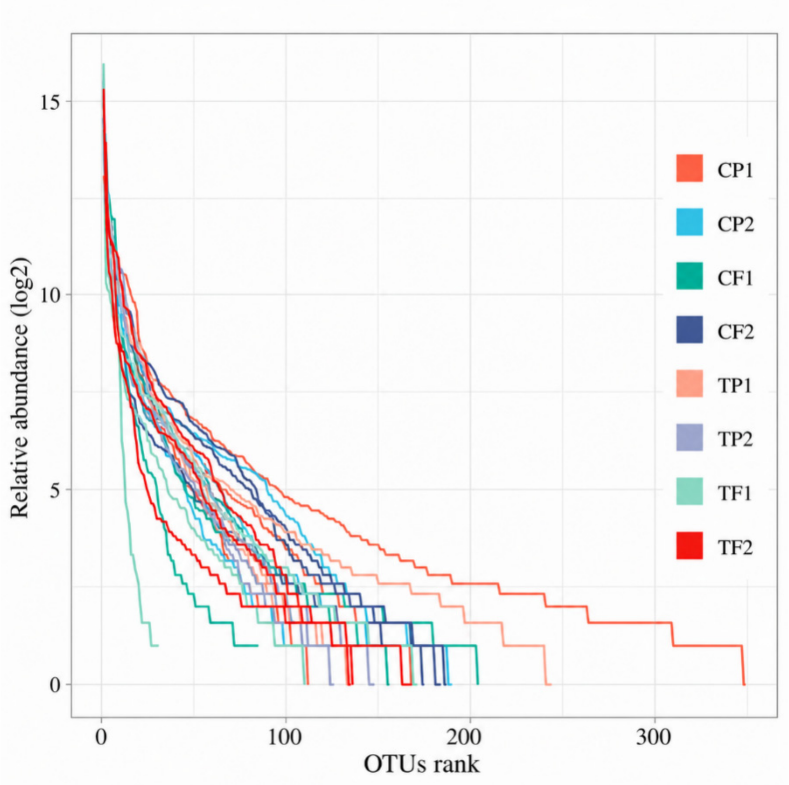

Figure S1. Rank abundance curves of 24 samples (merged into 8 groups). C: control (normal light: 8000 lx); T: UV stress (simultaneous UVA365 200 µW/cm² and UVB297 30 µW/cm² for 3 days); P: *Porites* sp. mucus; F: *Favites* sp. mucus; the numbers 1 and 2 mean two individuals of the same coral species, three parallel samples of the same individual of the same coral species, i.e. totally 24 sequencing samples are included for two species of corals.

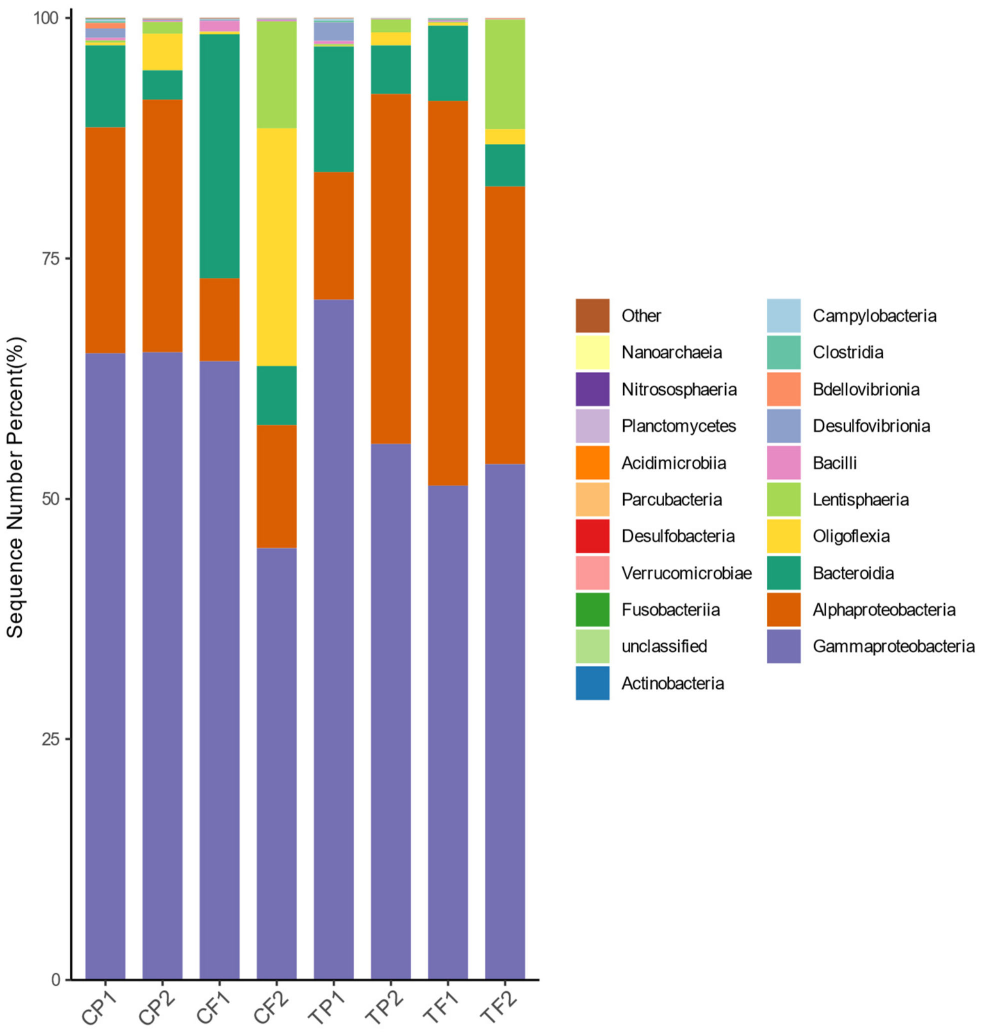

Figure S2. Class-level analysis of two types of coral mucus under UV stress (simultaneous UVA365 200 μW/cm² and UVB297 30 μW/cm² for 3 days) compared to the control (normal light: 8000 lx). C: control; T: represents UV stress; P: *Porites* sp. mucus; F: *Favites* sp. mucus; the numbers 1 and 2 mean different individuals of the same coral species. The numbers 1 and 2 mean two individuals of the same coral species, three parallel samples of the same individual of the same coral species, i.e. totally 24 sequencing samples are included for two species of corals.

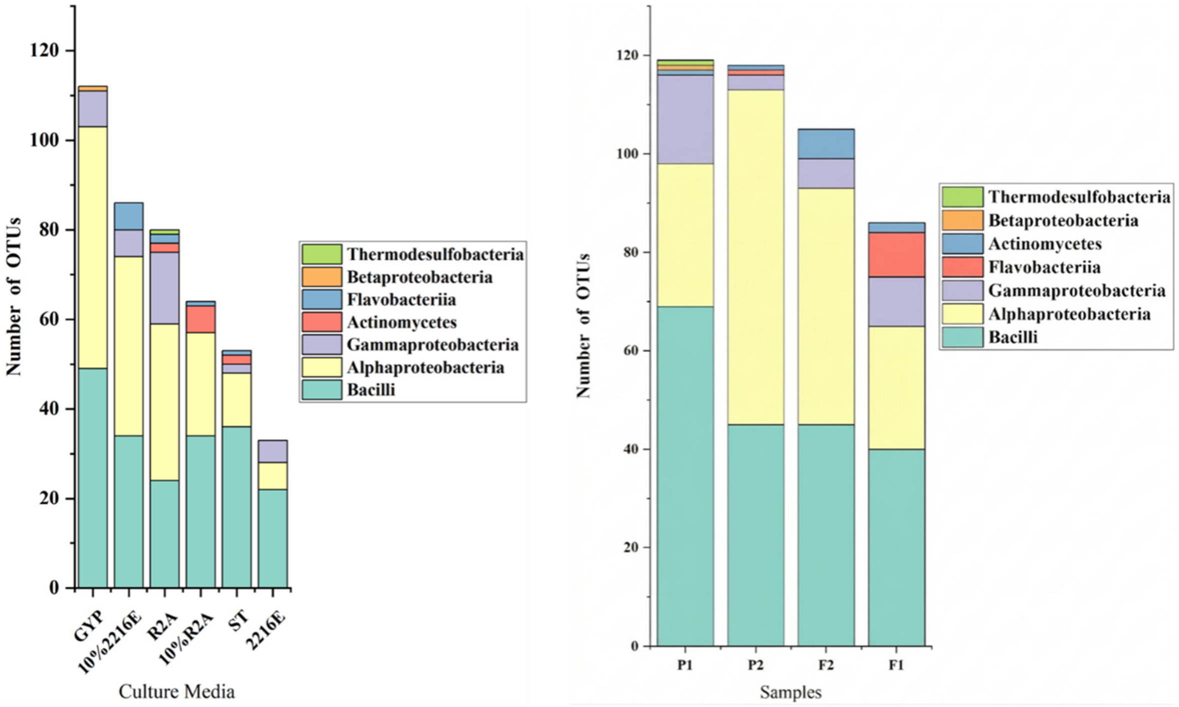

A B

Figure S3. Diversity distribution of cultivable bacteria at the class level. (A) Distribution based on different culture media; (B) Distribution based on different coral host samples. P: *Porites* sp. mucus; F: *Favites* sp. mucus. The numbers 1 and 2 mean two individuals of the same coral species with three parallel samples of the same individual of the same coral species.
